# A systematic screen reveals new elements acting at the G2/M cell cycle control

**DOI:** 10.1186/gb-2012-13-5-r36

**Published:** 2012-05-24

**Authors:** Francisco J Navarro, Paul Nurse

**Affiliations:** 1 Cell Cycle Lab. Cancer Research UK-London Research Institute, Lincoln's Inn Fields 44, London WC2A 3LY, UK; 2 Laboratory of Yeast Genetics and Cell Biology, The Rockefeller University, York Avenue 1230, New York 10065, USA; 3 Francis Crick Institute, Euston Road 215, London, NW1 2BE, UK

## Abstract

**Background:**

The major cell cycle control acting at the G2 to mitosis transition is triggered in all eukaryotes by cyclin-dependent kinases (CDKs). In the fission yeast *Schizosaccharomyces pombe *the activation of the G2/M CDK is regulated primarily by dephosphorylation of the conserved residue Tyr15 in response to the stress-nutritional response and cell geometry sensing pathways. To obtain a more complete view of the G2/M control we have screened systematically for gene deletions that advance cells prematurely into mitosis.

**Results:**

A screen of 82% of fission yeast non-essential genes, comprising approximately 3,000 gene deletion mutants, identified 18 genes that act negatively at mitotic entry, 7 of which have not been previously described as cell cycle regulators. Eleven of the 18 genes function through the stress response and cell geometry sensing pathways, both of which act through CDK Tyr15 phosphorylation, and 4 of the remaining genes regulate the G2/M transition by inputs from hitherto unknown pathways. Three genes act independently of CDK Tyr15 phosphorylation and define additional uncharacterized molecular control mechanisms.

**Conclusions:**

Despite extensive investigation of the G2/M control, our work has revealed new components of characterized pathways that regulate CDK Tyr15 phosphorylation and new components of novel mechanisms controlling mitotic entry.

## Background

An important aspect of the eukaryotic cell cycle control is the co-ordination of cell cycle progression with the growth of the cell. The investigation of this problem, extensively studied in the yeasts *Saccharomyces cerevisiae *and *Schizosaccharomyces pombe*, elucidated the basic molecular mechanisms of cell cycle control, which in many aspects are common to all eukaryotes. Genetic studies in the yeasts revealed that this co-ordination occurs at both the G1/S and the G2/M transitions, with G1/S being the major point of control for *S. cerevisiae *and G2/M for *S. pombe *[1,2]. Useful mutants for defining genes involved in the rate limiting steps of these transitions are those that advance cells prematurely into cell division, resulting in cells with a smaller cell size than normal [3,4]. The first of these mutants in fission yeast, *wee1-50*, was defective in a protein kinase that phosphorylates Tyr15 of the cyclin-dependent kinase (CDK) Cdc2 [5,6]. Phosphorylation of this conserved residue inhibits the CDK, and its dephosphorylation by the phosphatase Cdc25 activates the CDK and triggers mitosis [7-9]. This posttranslational modification is the major rate-limiting control of mitotic onset in fission yeast. Two pathways, the mitogen-activated protein kinases stress-nutritional response (SR) and the cell geometry sensing (CGS) pathways, regulate Tyr15 phosphorylation upstream of Wee1 and Cdc25 [10-15]. The SR pathway connects the nutrient-responding target of rapamycin (TOR) pathway to the recruitment of Polo kinase to the spindle pole body and CDK activation [15,16]. This pathway is responsible for nutritional modulation of mitotic entry. The other pathway that controls mitotic entry is formed by the Cdr1 and Cdr2 kinases, which regulate Wee1 activity in response to cell geometry, and involves a gradient of the protein kinase Pom1 along the long axis of the cell [13,14,17].

Tyr15 phosphorylation is considered the major regulatory mechanism of the G2/M transition in fission yeast. However, the observation that cells driven by a simplified cell cycle system lacking this control are still able to divide and coordinate cell division with mass increase suggests the existence of additional regulatory mechanisms [18]. The availability of near genome-wide collections of gene deletions provides an exceptional tool for systematically identifying components of the pathways that regulate the G2/M transition.

In this work we have screened the *S. pombe *gene deletion collection for mutants that prematurely enter into mitosis. We found 18 genes that function as negative regulators of mitosis, 7 of which have not been associated with cell cycle control before. Further analysis of these mutants identified putative new elements that regulate the G2/M transition acting upstream of the SR and CGS pathways. Additionally, we found genes that regulate the G2/M transition independently of Tyr15 phosphorylation, defining new rate limiting controls for mitotic entry. Therefore, our work provides a more complete view of the regulatory mechanisms acting at the G2/M transition.

## Results and discussion

### Systematic screen for small cell size mutants

Given the importance of the G2/M transition for cell cycle control, we have screened a near genome-wide fission yeast gene deletion collection [19] to search systematically for gene deletion mutants that divide prematurely, with the objectives of characterizing more comprehensively the components and mechanisms acting in a negative manner at the G2/M control.

**Figure 1:**
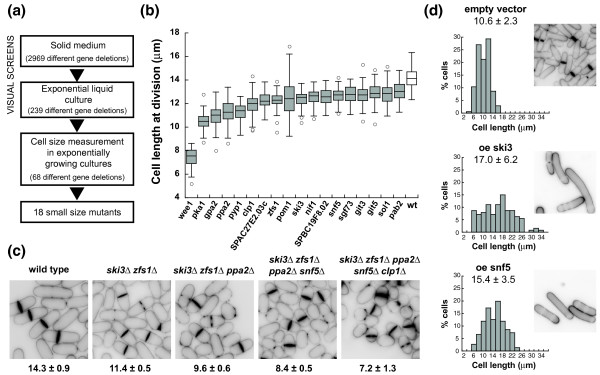
**Identification of small size mutants**. **(a) **Strategy for the identification of small size mutants in the fission yeast deletion collection. Microcolonies of 2,969 strains carrying individual deletions of different non-essential genes were visually screened on agar plates of yeast extract complex medium (YE4S). Candidate strains were grown in YE4S liquid exponential culture and their cell size determined in a secondary visual screen. Cell length and width of septated cells were measured for each of the selected strains. Gene deletions were confirmed by PCR, and co-segregation of the phenotype with the gene marker used in the deletion was checked. **(b) **Cell length at division of the small size mutants growing exponentially in complex medium. Boxes enclose 50% of the data and lines within the box show median cell length. Whiskers mark maximum and minimum values that fall within 1.5 standard deviations. Values outside this range are displayed as dots (n = 60 cells). Wt, wild type. **(c) **Dividing cells carrying additive combinations of mutations identified in the screen. Cell wall was stained with Blankophor and cell length at division in microns is indicated under each panel. **(d) **Length of cells carrying empty vector and overexpressing *snf5 *or *ski3*. Left panels: cell length distribution of the whole population after 20 h of overexpression. Values correspond to the average cell length in microns of the whole population and standard deviation (n > 125 cells). Right panels: Blankophor-stained cells after 20 h of overexpression.

We screened 82% of all fission yeast non-essential genes for mutants dividing prematurely at a small cell size, but with minimal effects on growth to avoid mutations influencing cell size indirectly [20]. The screening procedure is summarized in Figure 1a and consisted of an initial microscopic visual screen followed by length and width measurements at cell division of candidate mutants (see Material and methods). Fission yeast cells grow by linear extension and therefore cell length correlates with cell volume, facilitating the identification of a relatively subtle size phenotype. We identified 18 mutants that divided at least 1 μ;m shorter than the wild-type strain, which, under the growth conditions used, divided at a length of 14.1 μ;m (Figure 1b; Table 1; Additional file 1; Figure S1 in Additional file 2). We confirmed that cell volume, calculated from length and width at division, was also reduced in the selected mutants (Table 1). The smallest mutant found was *wee1*Δ, which divided at 7.4 μ;m, around half the cell length of the control strain. The rest of the mutants divided with cell lengths of 75 to 93% of the control strain. In the course of our screen we also observed mutants with significant heterogeneity in cell size at division due to the presence of longer cells. Because these long cells could have arisen from a transient arrest of the cell cycle or delayed mitosis, they were not studied further.

**Table 1:** Small size mutants identified in the genome-wide screen in fission yeast

Gene	Cell length at division^a ^(μm)	CV^b ^(%)	Cell width^c ^(μm)	Cell volume^d ^(μm^3^)	Doubling time^e ^(min)	Molecular function	Biological process	*S. cerevisiae *orthologs
*wee1*	7.4 ± 0.7	9.5	4.1 ± 0.2	80.5 ± 12.6	214 ± 10.4^f^	Protein kinase	G2/M transition	*SWE1*
*pka1*	10.5 ± 0.7	6.7	4.7 ± 0.2	153 ± 20.8	159 ± 2.3	cAMP-dependent protein kinase	Glucose sensing/cAMP signaling	*TPK1*, *TPK2*, *TPK3*
*gpa2*	10.9 ± 0.9	8.3	4.5 ± 0.3	151.9 ± 18	181 ± 1.3	G protein, α-subunit		*GPA2*
*git5*	12.9 ± 0.9	7.0	4.5 ± 0.2	179.6 ± 17.3	142 ± 4.9	G protein, β-subunit		*STE4*
*git3*	12.7 ± 0.8	6.3	4.6 ±0.2	183.9 ± 24.8	145 ± 3.8	G-protein coupled receptor		*GPR1*
*zfs1*	12.3 ± 0.7	5.7	4.1± 0.2	142.7 ± 13.6	140 ± 4.1	RNA binding	mRNA degradation	*CTH1*, *TIS11*
***ski3***	12.4 ± 0.6	4.8	4.3 ± 0.2	162.2 ± 15.1	135 ± 1.6	SKI complex component		*SKI3*
*clp1*	11.9 ± 0.9	7.6	4.4 ± 0.2	157.7 ± 15.8	138 ± 0.7	Protein phosphatase	G2/M transition cytokinesis	*CDC14*
*pom1*	12.3 ± 1.6	13.0	4.5 ± 0.2	174.1 ± 31.1	141 ± 5.3	Protein kinase		
*nif1*	12.5 ± 0.7	5.6	4.3 ± 0.2	159.6 ± 15.2	128 ± 2.3	Protein kinase inhibitor	G2/M transition	*DSF2*
***snf5***	12.6 ± 0.7	5.6	4.3 ± 0.2	163.8 ± 15.9	135 ± 1.6	SWI/SNF subunit	Chromatin remodeling	*SNF5*
***sol1***	12.9 ± 0.9	7.0	4.3 ± 0.2	162.9 ± 19.6	135 ± 4.4	SWI/SNF subunit		*SWI1*
***sgf73***	12.7 ± 0.8	6.3	4.2 ± 0.3	157.0 ± 20.9	159 ± 5.1	SAGA subunit	Histone modification	*SGF73*
***pab2***	13.1 ± 0.8	6.1	4.3 ± 0.2	165.7 ± 19.3	143 ± 4.7	RNA binding	mRNA poly(A) tail length	*SGN1*
**SPBC19F8.02**	12.6 ± 0.6	4.8	4.3 ± 0.2	160.6 ± 14.1	134 ± 1.5	NA	NA	
**SPAC27E2.03c**	12.3 ± 0.7	5.7	4.3 ± 0.2	160.3 ± 18.4	157 ± 6.1	GTP binding	NA	*OLA1*
*ppa2*	11.2 ± 1.0	8.9	4.3 ± 0.2	139.7 ± 15.9	136 ± 1.2	Protein phosphatase	Signal transduction	*PPH21*, *PPH22*
*pyp1*	11.3 ± 0.6	5.3	4.7 ± 0.2	166.2 ± 15.8	143 ± 4.7	Protein phosphatase	Stress-response MAPK cascade	*PTP2*, *PTP3*
WT	14.1 ± 0.8	5.7	4.5 ± 0.2	199.4 ± 21.8	129 ± 2.2			

Genes in bold are those that have not been previously implicated in cell cycle control. ^a^Mean ± standard deviation (SD), n = 60. ^b^Coefficient of variance. ^c^Cell width was measured at the center of the cell. Mean values ± SD (n = 60). ^d^Cell volume was estimated assuming *S. pombe *cell shape as a cylinder topped with two hemispheres (V = (4/3) × π × (Width/2)^3 ^+ π × (Width/2)^2 ^× (Length - Width)). Mean values ± SD (n = 60). ^e^Mean ± standard error (n = 3). ^f^The doubling time for *wee1*Δ was longer than that reported for the temperature sensitive *wee1-50 *mutant at restrictive temperature [3]. This is likely due to the accumulation of diploid cells and cells with aberrant septa in the deletion mutant. MAPK, mitogen-activated protein kinase; NA, not available.

All mutants grew with doubling times essentially similar to wild type (Table 1), except for the *wee1*Δ and *gpa2*Δ strains, with doubling times 66% and 40% longer than the wild-type strain. All mutants showed cell cycle phase distributions similar to the wild-type strain (Figure S2 in Additional file 2) except for the *wee1*Δ mutant, which had an extended G1 phase as previously noted [21]. Deletions of five other genes (*ypa2*, SPAC227.01c, *sft1*, *cpp1 *and Lac1) showed cell sizes smaller than wild type but were not analyzed any further because of their sick and slow growing phenotype.

All 18 genes identified are conserved across eukaryotes and most can be grouped into four categories based on their biological functions: (1) regulation of the G2/M CDK activity and cytokinesis (*wee1*, *clp1*, *pom1*, *nif1*, *ppa2*, *pyp1*); (2) glucose sensing/cAMP signaling pathway (*pka1*, *gpa2*, *git5*, *git3*); (3) mRNA metabolism (*zfs1*, *ski3*, *pab2*); and (4) chromatin structure (*snf5*, *sol1*, *sgf73*). Other genes not found in these categories were SPAC27E2.03c and SPBC19F8.02, with unknown functions. Eleven of the genes identified have been previously reported to be involved in the G2/M control, validating our screen. We cannot give an estimate of the false negative rate of our screen, but it is informative that all gene deletions reported previously to significantly reduce cell size that were present in the set of mutants we screened were found in our study. Our list of mutants does not include several other loss-of-function mutations previously reported to divide at a small cell size. This was because these other mutant strains did not divide at a sufficiently small cell volume to attain the cutoff we used in our growth conditions (YE4S media, 32°C; Table S2 in Additional file 2). Interestingly, we found seven genes for which the small size phenotype has not been previously described; *ski3*, *snf5*, *sol1*, *sgf73*, *pab2*, SPBC19F8.02 and SPAC27E2.03c (Tables 1 and 2).

**Table 2:** Function of the genes identified on the G2/M control

Through CDK Tyr15 phosphorylation				Independent of	
		
	Upstream Sty1	Upstream Cdr1	Upstream Sty1, Cdr1	CDK Tyr15 phosphorylation	Not determined
*wee1*	*pyp1*	*pom1*	*nif1*	***snf5***	*ppa2*
*clp1*	*gpa2*		***ski3***	***sol1***	***sgf73***
	*pka1*			*zfs1*	
	*git3*				
	*git5*				
	***pab2***				
	**SPAC27E2.03c**				
	**SPBC19F8.02**				

Analysis of the genetic interaction of the genes identified indicates that most of them act on the G2/M transition through CDK Tyr15 phosphorylation, directly on the CDK (Wee1), or indirectly, through Cdc25 (Clp1), the stress-nutritional response pathway (Pyp1, Gpa2, Pka1, Git3, Git5, Pab2, SPAC27E2.03c, SPBC19F8.02), the cell geometry sensing pathway (Pom1) or through both these two latter pathways (Nif1 and Ski3). The role of Snf5, Sol1 and Zfs1 in the G2/M control is independent of CDK Tyr15 phosphorylation, CDK inhibitor Rum1 and CDK protein levels. Genes in bold are those not previously associated with cell cycle control.

Comparison of our results with the list of budding yeast small size mutants identified in [22,23] revealed only limited overlap confined to *gpa2*/*GPA2 *and *wee1*/*SWE1 *(Table S3 in Additional file 2). The SAGA complex involved in chromatin modification was also present in both lists but represented by different subunits (Sgf73 in fission yeast, Ada1 and Spt3 in budding yeast). The two budding yeast studies differ in the growth conditions used, as Jorgensen *et al*. [22] scored cell size of exponentially growing strains while Zhang *et al*. [23] determined cell size from cultures grown to saturation. We specifically examined the cell size phenotype of fission yeast mutants in ortholog genes of the budding yeast genes found in [22,23]. Thirty-seven genes were identified as fission yeast orthologs to the 45 budding yeast genes that result in small size when deleted, and 23 were contained in the set of mutant strains screened. Only four (*GPA2*/*gpa2*, *SWE1*/*wee1*, *SCH9/sck2 *and *SLT2/pmk1*) genes passed to the liquid screen and finally only *GPA2*/*gpa2 *and *SWE1*/*wee1 *showed a significant small cell size phenotype in both yeasts. Interestingly, none of the genes identified in our study are directly involved in ribosome biogenesis, which was the major pathway represented in the small size mutants found by Jorgensen *et al*. [22]. This was not because of a low representation of 'ribosome biogenesis' annotated genes in our set of mutant strains, since approximately a third of all *S. pombe *genes annotated to this Gene Ontology category were present in this set (103 out of 325). The absence of genes involved in ribosome biogenesis from our list of small size mutants could be due to the different strategies used for coordinating cell division with growth in the two organisms, which in budding yeast occurs at G1/S while in fission yeast is usually at G2/M [2]. It is possible that the G1/S control could be more sensitive to the ribosome biogenesis than the G2/M control. It is also possible that the small size phenotype of the budding yeast ribosome biogenesis gene mutants results as a response of the cell to the reduction in the growth rate in these mutants rather than to a direct involvement of these genes in cell mass-cell cycle coordination.

Most of the identified mutations had only modest effects on cell size, but we found that combining different mutations reduced cell length further. The quintuple mutant *ski3*Δ *zfs1*Δ *ppa2*Δ *snf5*Δ *clp1*Δ divided with a cell length of 7.2 μ;m, 50% smaller than the wild type (Figure 1c). The additive interaction between mutations regarding cell size suggests that these genes define different pathways regulating the G2/M transition. Furthermore, the heterozygous diploid strain *ski3^+^/ski3*Δ *zfs1^+^/zfs1*Δ *ppa2^+^/ppa2*Δ *snf5^+^/snf5*Δ *clp1^+^/clp1*Δ was 23% smaller than the control diploid strain (16.6 μ;m versus 21.4 μ;m), establishing that these genes have a quantitative effect on the G2/M transition. Additionally, it has been reported before that an increase in the levels of Wee1, Pka1, Ppa2, Pyp1, Clp1, Pom1 and Nif1 caused cell elongation, which is a sign of mitotic delay or arrest [5,14,24-28]. We tested whether the overexpression of any of the remaining genes identified in our screen (SPAC27E2.03c, *zfs1*, *ski3*, SPBC19F8.02, *snf5*, *sgf73*, *sol1 *and *pab2*) also caused cell elongation, and found that overexpression of *ski3 *and *snf5 *significantly increased cell size (Figure 1d), establishing that they act as gene dosage-dependent regulators of the G2/M transition.

### Novel elements of regulatory pathways of the G2/M transition

**Figure 2:**
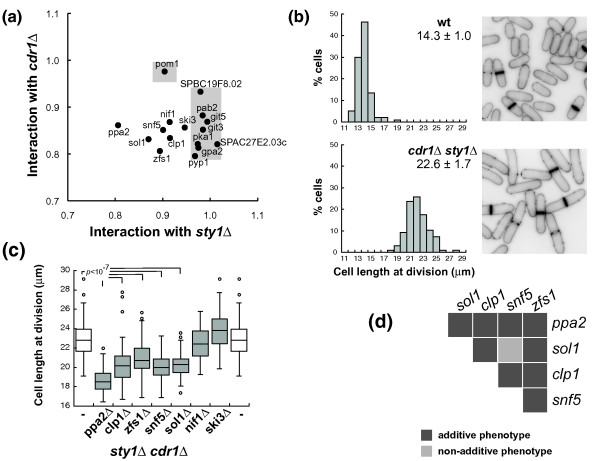
**Small size mutants are defective in the signaling of different G2/M regulatory pathways**. **(a) **Genetic interactions between identified mutations and the *cdr1 *and *sty1 *genes. Horizontal axis represents the ratio of cell length at division of the double mutant of each identified mutation in combination with *sty1*Δ over the single *sty1*Δ mutant. The vertical axis represents the cell length ratio of the double mutant with *cdr1*Δ over the single *cdr1*Δ mutant cell length. Shaded areas indicate genes that showed interaction with either *sty1*Δ or *cdr1*Δ. **(b) **Distribution of cell length at division and pictures of Blankophor-stained cells of the wild type (wt) and *cdr1*Δ *sty1*Δ double mutant strains. Mean cell length values ± standard deviation (n = 97) are indicated. **(c) **Cell length at division of triple mutants carrying *sty1*Δ and *cdr1*Δ deletions, and gene deletions that were additive to either *sty1*Δ or *cdr1*Δ. *P*-value was determined by Student's *t*-test. **(d) **Genetic interactions within the group of mutations that reduce cell size of the *cdr1*Δ *sty1*Δ double mutant. In the figure, additive phenotype means that the double is significantly smaller (*P *< 0.01) than both single parental strains.

We next investigated if the genes identified encoded components of the upstream pathways that regulate the activation of the G2/M CDK. For example, Pom1 and Pyp1 are respectively components of the CGS and the SR pathways [10,13,14]. We examined genetic interactions with the regulators Sty1 and Cdr1, which act at the base of each respective pathway (Figure 2a; Table S4 in Additional file 2). The plot in Figure 2a graphically summarizes our results. The *sgf73*Δ gene deletion in both *cdr1*Δ and *sty1*Δ backgrounds (Table S4 in Additional file 2), or in a double mutant *cdr1*Δ *sty1*Δ (Figure S3 in Additional file 2), reduced growth rate dramatically and resulted in cells with cytokinesis defects, so this gene was excluded from this analysis. All the remaining double mutants showed cell lengths similar to or smaller than *cdr1*Δ and *sty1*Δ single mutants. Approximately half the mutations tested (8 out of 17) did not reduce cell length of the *sty1*Δ mutant, indicating that the factors encoded by these genes function upstream of Sty1. This group is made up of Pyp1, Pab2, SPAC27E2.03c, SPBC19F8.02 and factors involved in glucose sensing-signaling, Git3, Git5, Gpa2 and Pka1. A connection between the glucose sensing/cAMP signaling pathway and Sty1 has previously been noted [29-31] and our work additionally establishes a key role for glucose sensing in the activation of the CDK. Conversely, all deletions reduced the size of the *cdr1*Δ strain except for *pom1*Δ as previously shown [13,14], indicating that Pom1 is the only component of the CGS pathway in our set of mutants. Interestingly, we also show that Nif1, which physically interacts with and inhibits Cdr1 [28], also appears to have a Cdr1-independent role in the G2/M transition.

The fact that a group of gene deletions (*ppa2*Δ, *sol1*Δ, *snf5*Δ, *zfs1*Δ, *clp1*Δ, *ski3*Δ, *nif1*Δ) reduced the cell size of both the *sty1*Δ and *cdr1*Δ strains indicated that these genes have roles in the G2/M control independently of these two pathways. To confirm the additive phenotype to both the *sty1 *and *cdr1 *gene deletions, we deleted these genes in a *sty1*Δ *cdr1*Δ strain. The double *sty1*Δ *cdr1*Δ mutant was viable and divided with a larger size than any of the parental mutants (Figure 2b; Table S5 in Additional file 2). Neither the *ski3 *nor *nif1 *deletion reduced cell length at division of the *cdr1*Δ *sty1*Δ mutant, suggesting that Ski3 and Nif1 function upstream of both Cdr1 and Sty1.

The *ppa2*, *sol1*, *snf5*, *zfs1 *and *clp1 *gene deletions reduced cell length at division of the *sty1*Δ *cdr1*Δ mutant (Figure 2c; Table S5 and Figure S4 in Additional file 2), confirming that their function in the G2/M is independent of both Sty1 and Cdr1. We investigated the genetic interactions within this group of genes (Figure 2d; Table S6 in Additional file 2) and found that, in all cases, mutants carrying pairs of deletions were smaller than the parental single mutant strains, with the one exception of the double mutant *snf5*Δ *sol1*Δ, which was similar to the *snf5*Δ alone. The additive genetic interactions within this group suggest that these genes function in different pathways. The non-additive *snf5*Δ *sol1*Δ result is consistent with the fact that Snf5 and Sol1 proteins are two subunits of the same complex [32]. Deletion of *snf22*, which encodes the ATPase subunit of this complex (not present in the deletions screened), also showed an advanced mitosis phenotype similar to the *snf5*Δ and *sol1*Δ mutants (cell length at division 12.7 ± 0.6 μ;m), confirming a role of the SWI/SNF complex in the G2/M control.

This analysis has revealed new components in the G2/M control that function upstream of Sty1 (Pab2, SPAC27E2.03c and SPBC19F8.02), has shown that Ski3 and Nif1 function through both Cdr1 and Sty1, and has identified other elements that function in the G2/M transition independently of the CGS and SR pathways (Ppa2, Sol1, Snf5, Zfs1 and Clp1).

### Tyr15 phosphorylation-independent regulation of the G2/M transition

**Figure 3:**
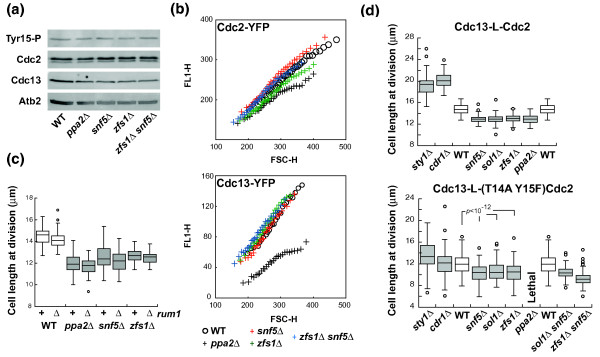
**Premature cell division of *snf5*Δ, *sol1*Δ and *zfs1*Δ does not result from alteration of CDK protein levels, Rum1 activity or CDK Tyr15 phosphorylation**. **(a) **Western blot of total cell lysates for phosphorylated-Tyr15 Cdc2, Cdc2, Cdc13 and α-tubulin (Atb2) protein levels in different small size mutants. **(b) **Cdc2-YFP (upper panel) or Cdc13-YFP (bottom panel) fluorescent signal in different small size mutants. Fluorescence signal of >7,000 cells was measured by flow cytometry and plotted as a moving average (window 250 cells) against forward scattered light. Note the reduced Cdc13-YFP level in the *ppa2*Δ strain. **(c) **Cell length at division of small size mutants carrying *rum1^+ ^*or *rum1*Δ gene deletion (n = 60 cells). **(d) **Size phenotype of gene deletions in a strain carrying the Cdc13-L-Cdc2 (upper panel) or Cdc13-L-(T14A Y15F)Cdc2 (bottom panel) fusion proteins. Upper panel, n = 60 cells; bottom panel, n = 150 cells. *P*-value was determined by Student's *t*-test. WT, wild type; YFP, yellow fluorescent protein.

We next investigated how *ppa2*, *sol1*, *snf5*, *zfs1 *and *clp1 *act at the G2/M transition. It is known that Clp1 regulates Cdc25 stability and consequently CDK Tyr15 phosphorylation [33,34]. We tested if the other genes of this group also had a role in Tyr15 phosphorylation or in other aspects of CDK activation. We first analyzed if CDK protein levels were altered. It is known that co-overexpression of the mitotic cyclin Cdc13 and CDK Cdc2 advances cells into mitosis [35]. However, the levels of Cdc13 and Cdc2 proteins determined both by western blot (Figure 3a) and by single cell fluorescence-activated cell sorting (FACS) analysis (Figure 3b) in the *ppa2*Δ, *snf5*Δ and *zfs1*Δ mutants, and in the double mutant *snf5*Δ *zfs1*Δ were similar to or lower than in the control strain. Therefore, the mitotic advancement observed in these mutants cannot be the result of an increase in CDK protein level. We also tested if the effects of these genes on the G2/M transition involve the CDK stoichiometric inhibitor Rum1, which inhibits the CDK during G1 [36,37]. Mutants carrying the *rum1 *deletion and the *zfs1*, *ppa2 *or *snf5 *deletions were viable, and the lengths at division were similar to the corresponding single mutants (Figure 3c). Therefore, the effects of *snf5*, *zfs1 *and *ppa2 *on the G2/M transition do not act through Rum1.

Finally, we investigated if these genes alter the phosphorylation levels of Cdc2 at residue Tyr15. The levels of phosphorylated Cdc2 in *ppa2*Δ, *snf5*Δ, *zfs1*Δ and the double mutant *snf5*Δ *zfs1*Δ were similar to those in the wild-type strain (Figure 3a), suggesting a role in the G2/M transition independent of Tyr15 phosphorylation regulation. To further support this observation, we tested if the effect of these gene deletions was also observed in a background containing a non-phosphorylatable Cdc2 mutant protein. We used a strain expressing a mutant Thr14Ala Tyr15Phe Cdc2 kinase (Thr14 is occasionally phosphorylated [38]) fused to the cyclin Cdc13, which is well tolerated by the cell [18] contrary to the non-fused mutant CDK [8]. Cells with this Cdc13-L-Cdc2 fusion protein have a wild-type doubling time, cell length and cell cycle distribution. In agreement with the roles of the SR and CGS pathways regulating the G2/M transition through CDK Tyr15 phosphorylation, the non-phosphorylatable CDK fusion protein and not the wild-type fusion protein specifically abolished most of the effects on mitotic onset of *sty1 *and *cdr1 *gene deletions (Figure 3d; Table S7 and Figure S5 in Additional file 2), establishing that this system can be used for testing if Snf5, Sol1, Ppa2 and Zfs1 act on the G2/M control through CDK Tyr15 phosphorylation. We deleted these genes individually in strains with Cdc13-L-Cdc2 or Cdc13-L-(T14A Y15F)Cdc2 fusion proteins. Deletions reduced cell length at division of the strain carrying the Cdc13-L-Cdc2 fusion protein in a similar way to that observed in the wild-type background (Figure 3d, upper panel; Table S7 and Figure S6a in Additional file 2). The deletion of *ppa2 *in the Cdc13-L-(T14A Y15F)Cdc2 background rendered cells inviable, similar to the lethal phenotype of the double mutant *wee1-50 ppa2*Δ at restrictive temperature [25]. We measured cell length at division of the remaining viable strains and found that cells harboring these deletions were shorter than the control strain, although the CDK could not be phosphorylated on Tyr15 (Figure 3d, bottom panel; Table S7 and Figure S6b in Additional file 2). The *snf5*Δ and *sol1*Δ deletions were not additive in the Cdc13-L-(T14A Y15F)Cdc2 background, while *snf5*Δ and *zfs1*Δ were additive, reducing cell length by 23%. These results show that the premature mitosis of *snf5*Δ, *sol1*Δ and *zfs1*Δ mutants is independent of Tyr15 phosphorylation and establishes that there must be additional regulatory mechanisms acting at the G2/M transition.

This systematic screen of more than 80% of fission yeast non-essential genes has identified a significant proportion of the genes acting negatively at the G2/M transition. The 18 genes identified are listed in Table 2 together with their connection to the G2/M control. We found that most of these genes function through CDK Tyr15 phosphorylation. Eight of these genes function upstream of *sty1*, and of these, three, *pab2*, SPAC27E2.03c and SPBC19F8.02, are described here for the first time as negative regulators of mitotic onset and define new components of the SR pathway. Only one gene, *pom1*, acts solely in the CGS pathway. However, our data indicate that *ski3 *and *nif1 *function in both the SR and CGS pathways, suggesting a cross-talk between these two pathways previously thought to act independently.

We found that *snf5*, *sol1*, *zfs1*, *ppa2 *and *clp1 *function independently of both *sty1 *and *cdr1*, and that *snf5*, *sol1 *and *zfs1 *act on mitotic onset independently of CDK Tyr15 phosphorylation. The advanced mitotic phenotype of their deletions, described for first time for *snf5 *and *sol1*, was not due to changes in CDK protein level or Rum1 deregulation, indicating that they represent components of uncharacterized rate-limiting controls acting at the G2/M transition. We suggest that the lethality of *ppa2*Δ when combined with the Tyr15 mutant CDK could be due to a role in the G2/M transition also independent of Tyr15 phosphorylation. These proteins could be involved in regulating the dephosphorylation of CDK substrates given that, in *Xenopus laevis *eggs, PP2A phosphatase controls cell cycle progression by counteracting the CDK-dependent phosphorylation of mitotic substrates [39], and in *S. cerevisiae*, Cdc14 (homolog of Clp1) dephosphorylates CDK substrates [40]. Alternatively, these proteins could function in some other way on CDK targets or could inhibit the CDK by unknown regulatory pathways. However, they would not be expected to have a role in the assembly of the CDK complexes given they still exert effects in the presence of the fusion protein Cdc13-L-Cdc2 (Figure 3d; Table S7 and Figure S6b in Additional file 2). Alternatively, these proteins might be involved in the cellular localization of the complex and their absence could facilitate access of the CDK to its substrates. Another possibility is that these proteins are involved in an as yet uncharacterized posttranslational modification of the CDK. The elucidation of the molecular details of the mechanism of action of these proteins on the G2/M transition will require further study.

## Conclusions

Much emphasis has been put on Cdc2 Tyr phosphorylation as the regulatory mechanism that ensures the coordination between cell growth and cell division. However, the fact that a synthetic CDK lacking the regulatory phosphorylation site still exhibits a significant degree of cell size homeostasis [18] argues strongly for the existence of other layers of regulation. Furthermore, we have shown here regulation of mitotic onset without involving CDK Tyr15 phosphorylation. Our work has identified new components of characterized pathways and has revealed the existence of new regulatory mechanisms, and therefore provides a more complete view of the regulatory network of G2/M control.

## Materials and methods

### Strains and growth conditions

*S. pombe *media and methods are described in Moreno *et al*. [41]. Strains used are listed in Table S8 in Additional file 2. Experiments were carried out in yeast extract complex media supplemented with 0.15 mg/ml L-Histidine, L-Leucine, Adenine and Uridine (YE4S) at 32°C.

### Screen for small size mutants

The viable set of a near genome-wide *S. pombe *haploid deletion collection [19] was screened for mutants that divide prematurely with a smaller size than the wild-type strain. In total, we screened the cell size phenotype of 2,969 different gene deletions (82% of fission yeast non-essential genes). Our strategy consisted of an initial microscopic visual screen of mutants growing on agar plates of complex media (YE4S) followed by measurements of cell size at division of candidate mutants growing exponentially in liquid culture. Mutant strains were arrayed in 96-well plates and 150 μ;l of YE4S per well was inoculated and incubated at 32°C for >16 h with shaking. We then inoculated a solid YE4S media plate using a pin tool and incubated this plate for 12 to 20 h until small colonies (40 to 60 cells) formed. Each mutant strain was inoculated in quadruplicate and was compared with the control strain PN558 (*ade6-*M210 *leu1-32 ura4-D18 h+*) growing in the same plate. The visual screen for cell size phenotypes was carried out using a Zeiss Axioskop 40 microscope equipped with a 20×/0.4 NA objective and an additional 1.8× magnification. From this first screen, we selected 239 different mutant strains for a second screen in liquid culture, in which growth conditions were better controlled. Candidate mutants were grown in individual flasks containing 15 ml of YE4S media at 32°C, and cell size was screened when the culture was growing in exponential phase (0.35 to 0.45 OD_595_). Cell wall and septum were stained with Blankophor (1:100,000; MP Biochemicals, Solon, OH, USA) and cells were observed with a Zeiss Axioskop microscope, equipped with a QICam Fast camera and using a 63×/1.4 NA objective. We selected 68 mutant strains selected at this stage, which were grown again under the same conditions, and the width and length of dividing cells measured from pictures. We selected mutants that divided at least 1 μ;m shorter in cell length than the control strain. Wide mutants that showed reduced cell length but wild-type volume were discarded as these mutants are altered in morphology rather than in the control of the cell cycle. Finally, mutants were backcrossed with the wild-type strain to confirm the co-segregation of the phenotype with the deletion marker and to remove auxotrophies, and the specificity of the gene deletion was verified by PCR. Genes in our set of small size mutants were deleted for more than 91% of the ORF [19]. Cell size phenotypes of the new small size mutants identified in our screen have been annotated in PomBase [42].

### Cell length measurements and statistical methods

Cell length and width were measured from pictures of live Blankophor-stained cells using the PointPicker plug-in of ImageJ (National Institutes of Health). Cell volume was estimated from the length and width values by considering the shape of a fission yeast cell as a cylinder topped with two hemispheres. Average cell length and width values were determined from 60 to 150 cells, and comparison between strains was done using a two-tailed unpaired Student's *t*-test. In box-and-whisker plots, boxes are delimited by the first quartile, median and third quartile, and whiskers mark maximum and minimum values within a range of up to 1.5 standard deviations. Values outside this range are displayed as individual dots.

### Gene deletion and overexpression

Gene deletion was performed as in [43]. Gene targeting oligos were designed using the tool 'Gene deletion' from the Bähler Lab web site [44]. Overexpression of genes was done using the multicopy plasmid REP3X under the thiamine-repressible *nmt1 *gene promoter [45]. ORFs were amplified by PCR with specific oligos carrying restriction enzyme sites for cloning into REP3X. In overexpression experiments, cells were grown in synthetic media (EMM) containing 5 μ;g/ml thiamine and expression of the gene was induced upon thiamine removal by filtration and incubation in thiamine-free medium for 20 h.

### Protein extracts and western blots

Total protein extracts were prepared from 10^8 ^cells, collected by centrifugation and resuspended in the same volume of HB buffer (60 mM β-glycerol phosphate, 15 mM MgCl_2_, 15 mM EGTA, 1% (v/v) Triton X-100, 150 mM NaCl, 25 mM MOPS-NaOH pH7.2), containing protease inhibitor (1 mM phenylmethylsulfonyl fluoride (PMSF and Roche Complete Mini protease inhibitor cocktail) and phosphatase inhibitors (Roche PhosStop, Roche, Indianapolis, IN, USA). Cell suspensions were boiled for 5 minutes, and then transferred to a tube containing 1.2 ml of glass beads (0.4 mm; Sigma, St Louis, MO, USA). Cells were disrupted in a FastPrep cell disruptor (ThermoSavant, Waltham, MA, USA) for 3 × 20 s. HB buffer plus inhibitors (50 μ;l) was added and the crude extract was recovered and mixed with 5× sample buffer (20% (v/v) β-mercaptoethanol, 20% (w/v) SDS, 0.05% (w/v) bromophenol blue, 25% (v/v) glycerol, 300 mM Tris-HCl pH6.8). Finally, extracts were boiled for 5 minutes and centrifuged at 13,000 rpm for 1 minute. In western blots, Cdc13 was probed with rabbit polyclonal SP4 antibody [9] (1:3,000); Cdc2 with commercial rabbit polyclonal anti-PSTAIRE (1:250; Santa Cruz Biotech, Santa Cruz, CA, USA); phoshorylated Tyr15 Cdc2 with commercial rabbit polyclonal (1:500; Cell Signaling Technology, Danvers, MA, USA); and Atb2 with monoclonal TAT1 antibody (1:5,000; gift from K Gull [46]). Horse radish peroxidase-conjugated goat anti-mouse or goat anti-rabbit IgG (Pierce-Thermo, Waltham, MA, USA) were used at a dilution of 1:10,000 as secondary antibodies.

### Flow cytometry

DNA content per cell was determined from 10^4 ^cells that were fixed with 70% (v/v) ethanol and then washed with 1 ml 50 mM sodium citrate. Cells were resupended in 0.5 ml 50 mM sodium citrate containing 0.1 mg/ml RNase A and incubated at 37°C overnight. DNA was stained with 2 μ;g/ml propidium iodide and samples were sonicated before analysis in a BD FACSCalibur instrument. Single cell analysis of CDK protein levels was performed from strains expressing yellow fluorescent protein (YFP)-tagged Cdc13 or Cdc2 proteins under their native promoters. Cells were grown in YE4S at 32°C and 1 ml of culture at 0.2 OD_595 _(approximately 4 × 10^6 ^cells) was fixed with 1% (w/v) formaldehyde for 15 minutes; then cells were washed and resuspended in 1 ml phosphate-buffered saline. Cells were briefly sonicated prior to measuring fluorescence signal in a FACSCalibur instrument (BD Biosciences, San Jose, CA, USA) equipped with a 488 nm excitation laser and a 530 nm bandpass filter. Autofluorescence from a non-YFP tagged strain was subtracted from the YFP fluorescent signal.

## Abbreviations

CDK: cyclin-dependent kinase; CGS: cell geometry sensing; ORF: open reading frame; PCR: polymerase chain reaction; SR: stress-nutritional response; YFP: yellow fluorescent protein.

## Competing interests

The authors declare that they have no competing interests.

## Authors' contributions

FN and PN conceived and designed the study. FN performed the experiments. FN and PN discussed and interpreted results, and wrote the manuscript. Both authors read and approved the final manuscript.

## Supplementary Material

Additional file 1 **Table S1 - strains analyzed and selected in every step of the cell size screen**. Click here for file

Additional file 2 **Supplementary Figures S1 to S6 and Tables S2 to S8**. Figures S1 and S4 to S6: histograms of cell length at division of different size mutants. Figure S2: cell cycle distributions of the small cell size mutants identified. Figure S3: photomicrographs showing the cytokinetic defects of the *sgf73*Δ *sty1*Δ *cdr1*Δ triple mutant. Table S2: loss-of-function mutations reported previously to decrease cell length at division in fission yeast. Table S3: comparison of mutants obtained in this study with *S. cerevisiae *small size mutants. Tables S4 to S7: cell length measurements of size mutants. Table S8: yeast strains used in this study. Click here for file
