# Supplementary material for: A systematic screen reveals new elements acting at the G2/M cell cycle control
Source: Genome Biol. 2012 May 24;13(5):R36. doi: 10.1186/gb-2012-13-5-r36 (PMC3446289; doi:10.1186/gb-2012-13-5-r36)
Supplement: Additional file 2 — Supplementary Figures S1 to S6 and Tables S2 to S8. Figures S1 and S4 to S6: histograms of cell length at division of different size mutants. Figure S2: cell cycle distributions of the small cell size mutants identified. Figure S3: photomicrographs showing the cytokinetic defects of the sgf73Δ sty1Δ cdr1Δ triple mutant. Table S2: loss-of-function mutations reported previously to decrease cell length at division in fission yeast. Table S3: comparison of mutants obtained in this study with S. cerevisiae small size mutants. Tables S4 to S7: cell length measurements of size mutants. Table S8: yeast strains used in this study. [file gb-2012-13-5-r36-S2.PDF]

A systematic screen reveals new elements acting at the G2/M cell cycle control

Francisco J. Navarro[1, §] and Paul Nurse[1,2,3]

[1] Cell Cycle Lab. Cancer Research UK, London Research Institute, 44, Lincoln's Inn Fields, London WC2A 3LY UK
[2] Laboratory of Yeast Genetics and Cell Biology, The Rockefeller University, New York, NY, 10065, USA
[3] Francis Crick Institute, London, UK

[§] Corresponding author

Email addresses:

FJN: Francisco.Navarro@cancer.org.uk

PN: Paul.Nurse@cancer.org.uk

Additional_file_2.pdf:

Figure S1, S4 to S6: histograms of cell length at division of different size mutants.
Figure S2: cell cycle distributions of the small cell size mutants identified.
Figure S3: pictures of *sgf73Δ sty1Δ cdr1Δ* triple mutant.
Table S2: Loss-of-function mutations reported previously to decrease cell length at division.
Table S3: Comparison of mutants obtained in this study with *Saccharomyces cerevisiae* small size mutants.
Tables S4-S7: Cell length measurements of size mutants.
Table S8: Yeast strains used in this study.

Figure S1. Histograms of cell length at division of the small size mutants.
Cell length at division was determined from exponentially growing cultures (n=60 cells).

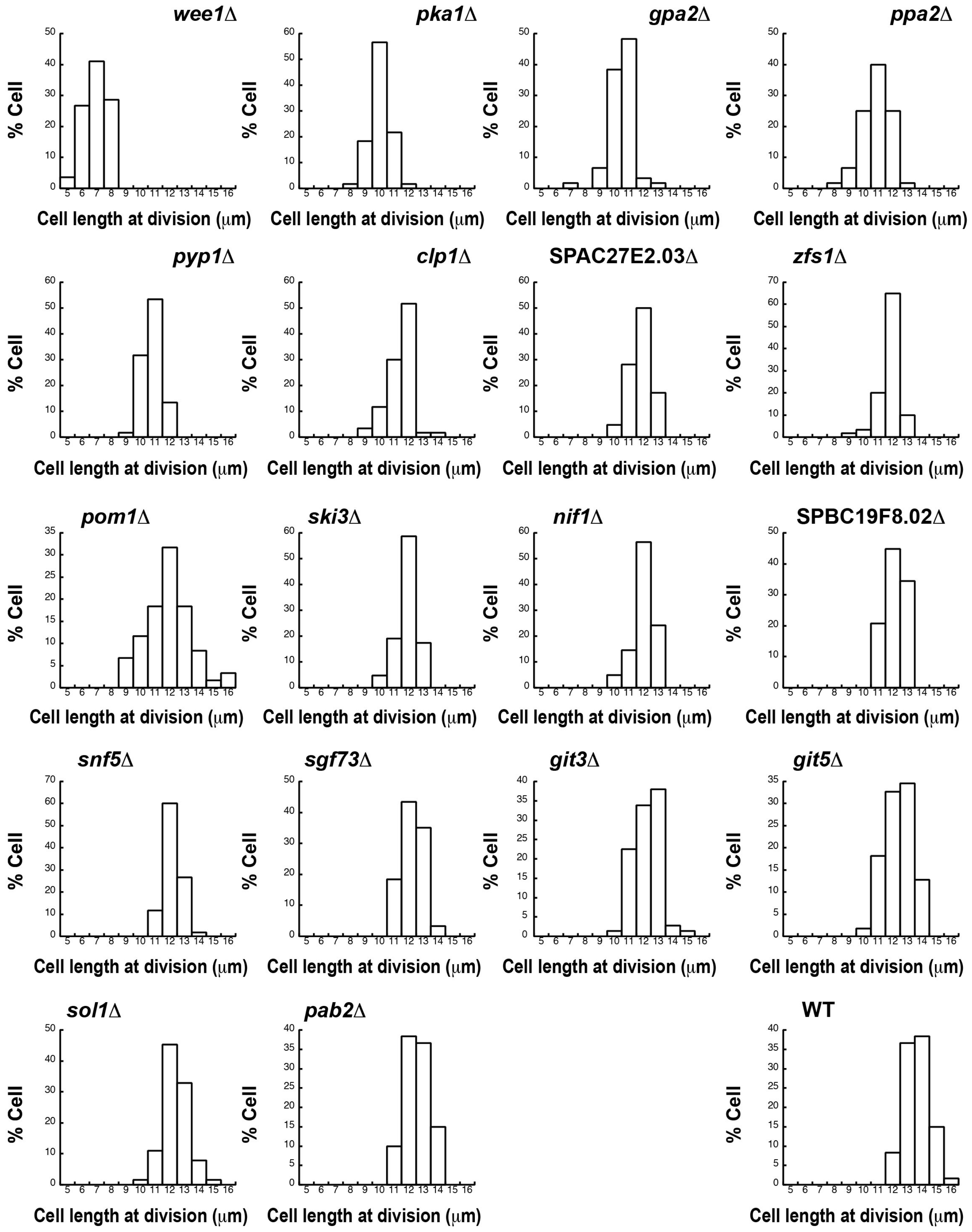

Figure S2. Cell cycle distribution of the small size mutants.
Histograms of DNA content/cell determined by FACS of asynchronous cultures growing exponentially.

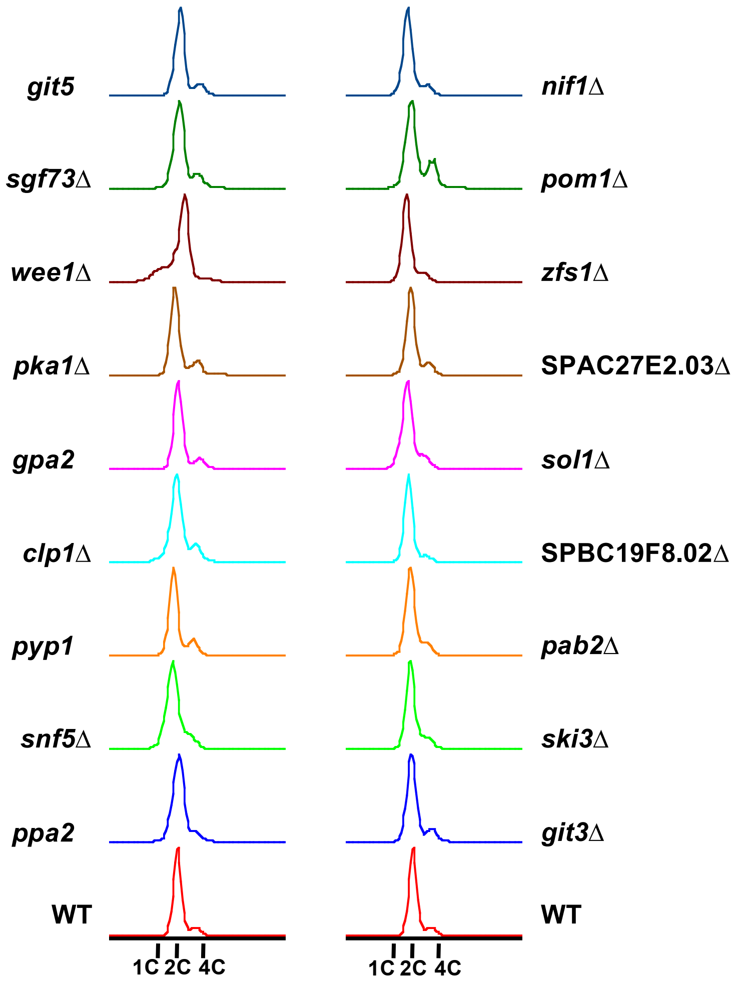

Figure S3. Deletion of *sgf73* gene in *sty1*Δ *cdr1*Δ double mutant results in cytokinesis defects.

Cells were grown to mid-logarithmic phase in complex media YE4S at 32°C. Cell wall and septum were stained with Blankophor. **A**, *sty1*Δ *cdr1*Δ strain; **B**, *sgf73*Δ *sty1*Δ *cdr1*Δ strain.

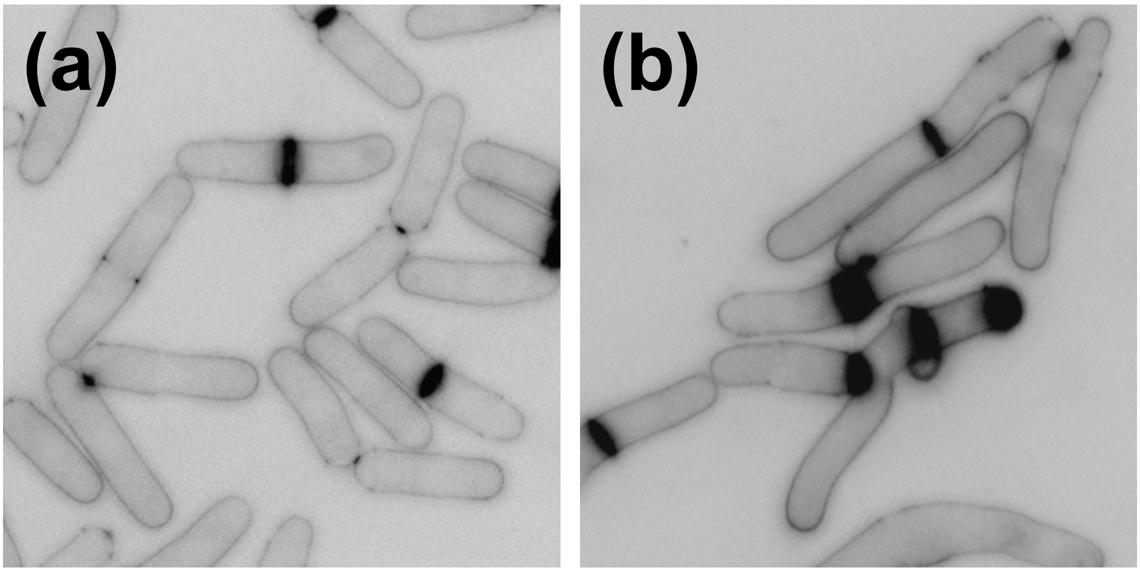

Figure S4. Histograms of cell length at division of mutant strains *sty1*Δ *cdr1*Δ. Cell length at division was determined from exponentially growing cultures (n=60 cells).

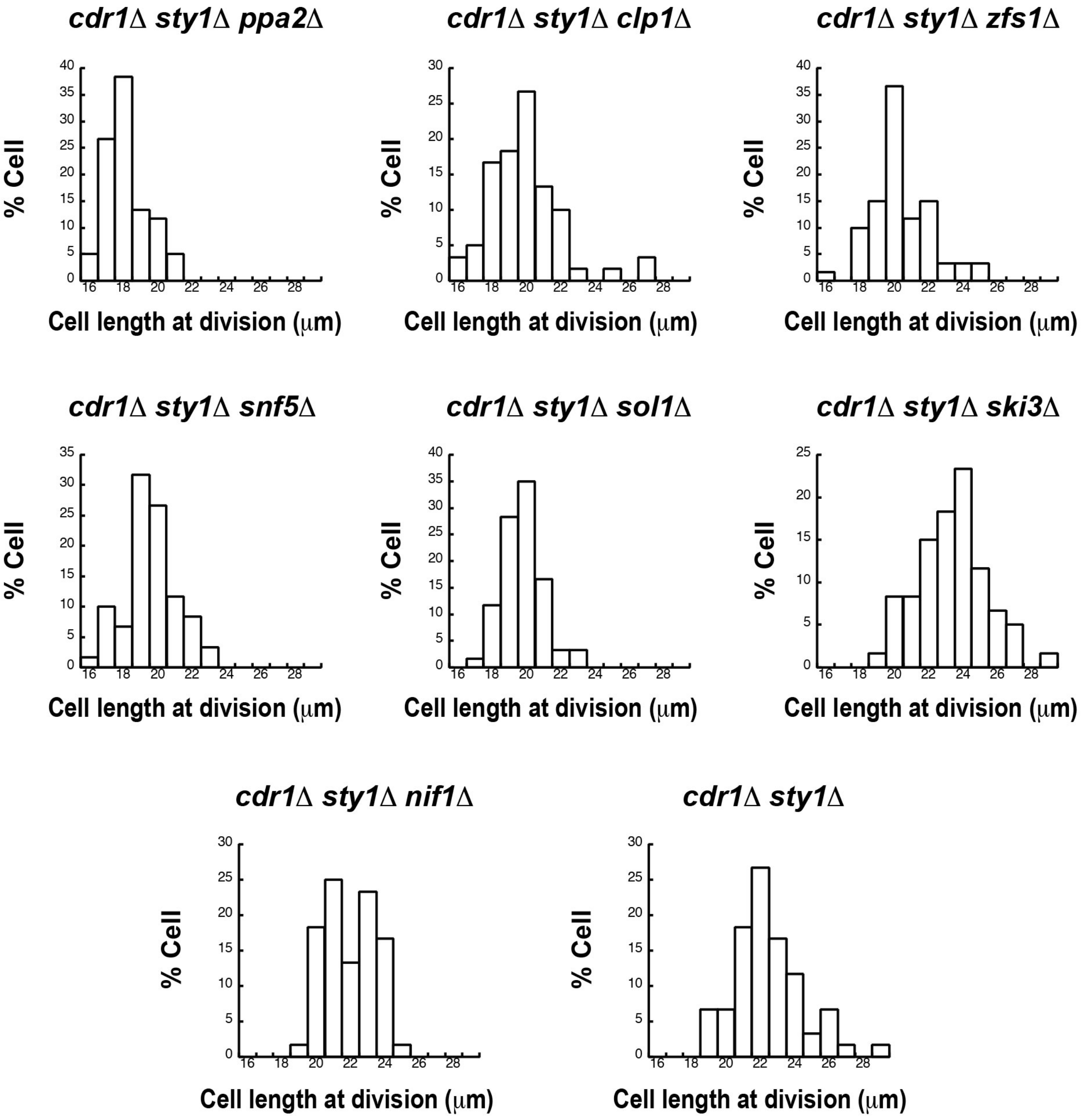

Figure S5. Histograms of cell length at division of mutant strains carrying a wild type Cdc13-L-Cdc2 or Cdc13-L-(T14A Y15F)Cdc2 fusion protein.

Cell length at division was determined from exponentially growing cultures (n=60 cells for Cdc13-L-Cdc2 strains; n=150 for Cdc13-L-(T14A Y15F)Cdc2 strains).

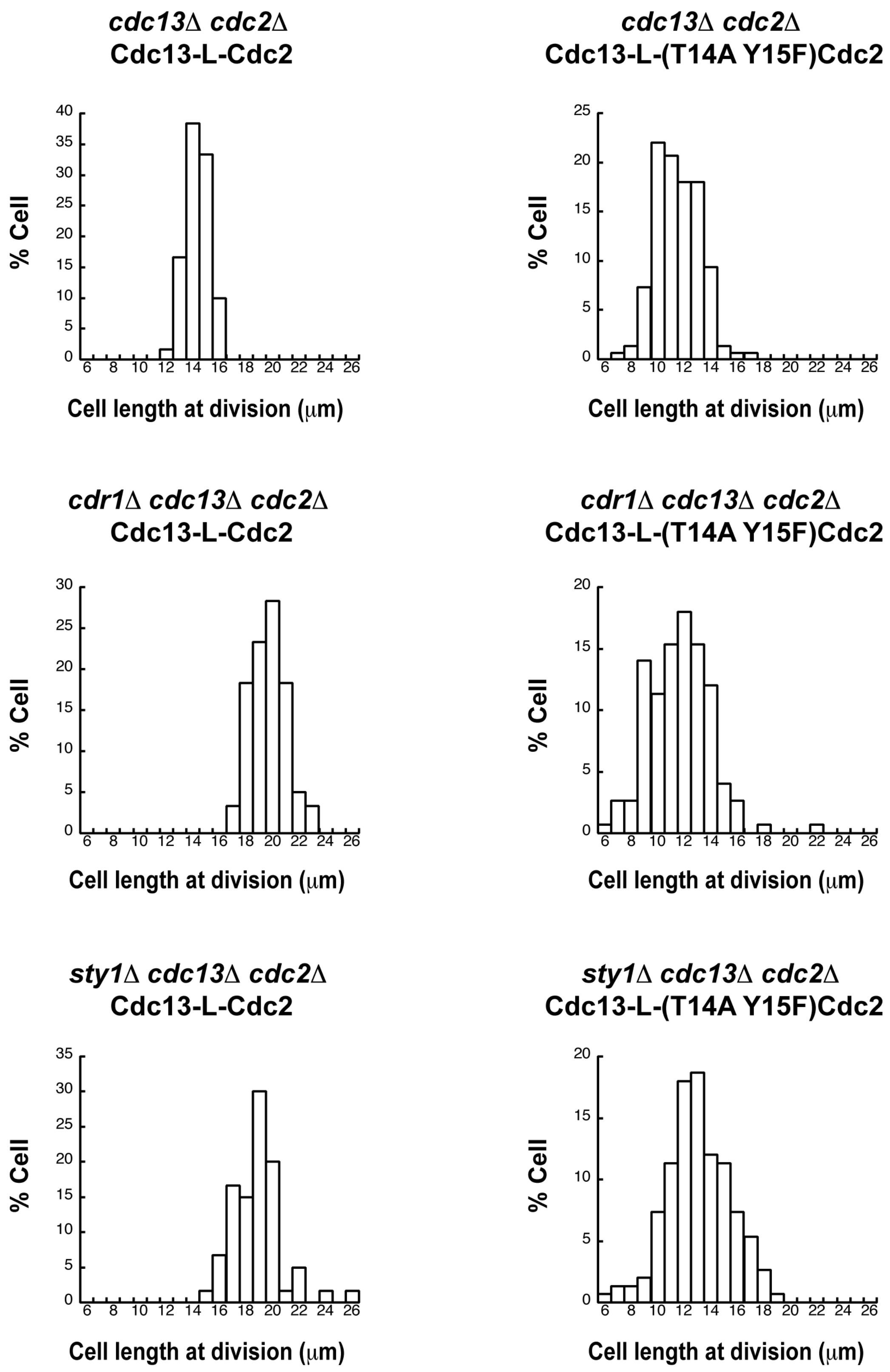

Figure S6. Histograms of cell length at division.
(a) Cell length distributions of strains carrying a Cdc13-L-Cdc2 fusion protein (n=60 cells). **(b)** Cell length distributions of strains carrying a Cdc13-L-(T14A Y15F)Cdc2 fusion protein (n=150 cells).

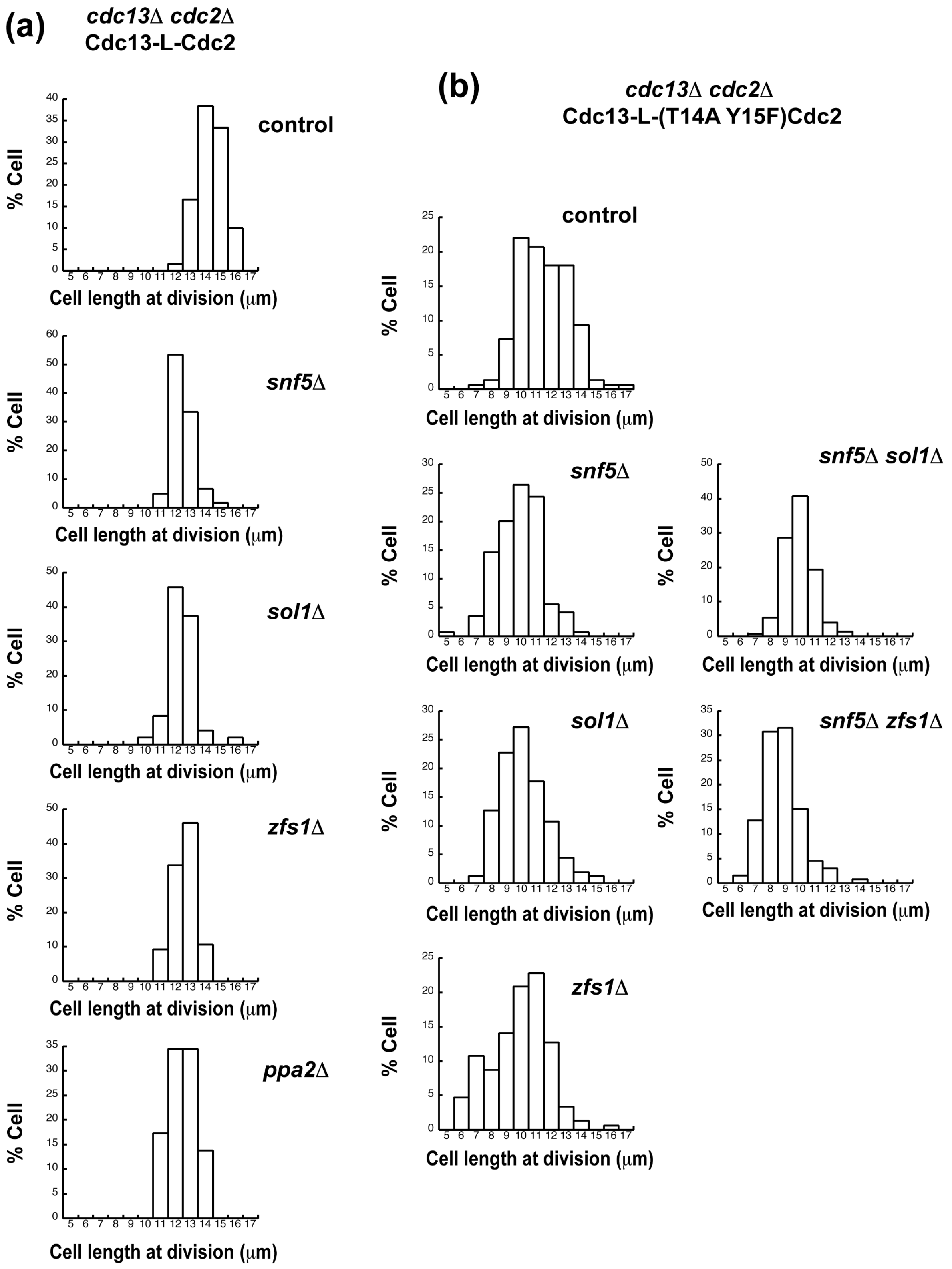

Table S2. Loss-of-function mutations previously reported to reduce cell size and which were not included in our list of genes.

Although present in our set of gene deletions, these mutants were not selected because they did not divide with a cell volume smaller than our cut-off under the conditions we used (YE4S media, 32°C). *rad24* and *pub1* mutants also showed a slow growing and sick phenotype.

Gene	Product	Biological function	Reference
SPY1	His-containing response regulator phosphotransferase	Stress-response cascade	[1]
PYP2	Tyr phosphatase	Stress-response cascade	[2]
SRK1	MAPK-activated protein kinase	Stress-response cascade	[3]
PUB1	HECT-type ubiquitin-protein ligase E3	Regulation of mitotic cell cycle, intracellular pH, transmembrane transport	[4]
RAD24	14-3-3 protein	Regulation of mitotic cell cycle, DNA damage checkpoint	[5]
RAD25	14-3-3 protein	Regulation of mitotic cell cycle, DNA damage checkpoint	[5]
TRA1	SAGA complex phosphatidylinositol pseudokinase	Histone acetylation, chromatin remodelling	[6]
SKB1	Type II protein Arg N-methyltransferase	Regulation of bipolar cell polarity, cellular response to osmotic stress	[7]
RRG1	Methyltransferase	Negative regulation of G2/M transition of mitotic cell cycle	[8]

Table S3. Comparison between fission yeast and budding yeast small size mutants.

S. cerevisiae small mutants list from Jorgensen *et al.* (2002) [9] and Zhang et al. (2002) [10]. In bold, common size regulators in both organisms.

Schizosaccharomyces pombe	***Saccharomyces cerevisiae***	
	Jorgensen *et al.* (2002)	Zhang et al. (2002)
wee1	***SWE1***	*ADA1*
gpa2	***GPA2***	*SPT3*
sgf73	*SFP1*	*WHI5*
pka1	*SCH9*	*MCK1*
git5	*PRS3*	*HAP2*
git3	*WHI3*	*SLT2*
zfs1	*WHI5*	*SDC25*
ski3	*RPA49*	*GAL80*
clp1	*JJJ1*	*HAP4*
pom1	*YNL226W*	*SAC1*
nif1	*PHO5*	*YKL037W*
snf5	*HXK2*	*RPL34B*
sol1	*PIH1*	*UBP15*
pab2	*SKY1*	*FMC1*
SPBC19F8.02	*KAP122*	*WHI2*
SPAC27E2.03	*SSF1*	*BCK1*
ppa2	*RPA14*	*KRH1*
pyp1	*YGR111W*	*SRM1*
	YCR061W	*SLG1*
	PTK2	*HTB1*
	TOM1	
	KEL1	
	YGR064W	
	CDH1	
	WHI6	

Table S4. Genetic interaction of small size mutants with the cell size regulators *sty1* and *cdr1*.

Mean cell length at division (in μm) and standard deviation (n=60) of single *sty1*Δ and *cdr1*Δ mutants (first row) and double mutants carrying either *sty1* or *cdr1* gene deletions plus each gene deletion found in the screen. ** indicates a *p*-value <0.01 determined by Student's t-test comparing means between single *cdr1*Δ or *sty1*Δ and double mutant.

	***sty1*Δ**	**%**		***cdr1*Δ**	**%**	
**-**	17.7 ± 1.2	100		17.5 ± 0.9	100	
***ppa2*Δ**	14.3 ± 1.0	80.8	**	15.1 ± 1.0	86.3	**
***sol1*Δ**	15.4 ± 0.9	87.0	**	14.5 ± 0.9	82.9	**
***snf5*Δ**	15.9 ± 0.9	89.8	**	14.9 ± 1.1	85.1	**
***zfs1*Δ**	15.8 ± 1.1	89.3	**	14.1 ± 0.9	80.6	**
***clp1*Δ**	16.2 ± 1.1	91.5	**	14.6 ± 1.1	83.4	**
***nif1*Δ**	16.2 ± 1.0	91.5	**	15.2 ± 0.9	86.9	**
***ski3*Δ**	16.8 ± 1.0	94.9	**	15.0 ± 1.0	85.7	**
***pom1*Δ**	16.0 ± 1.8	90.4	**	17.1 ± 1.6	97.7	
***sgf73*Δ**	18.7 ± 1.3	105.6	**	18.6 ± 1.4	106.3	**
**SPBC19F8.02Δ**	17.3 ± 1.1	97.7		16.3 ± 0.9	93.1	**
***pab2*Δ**	17.4 ± 2.4	98.3		15.4 ± 1.0	88.0	**
***git5*Δ**	17.6 ± 1.2	99.4		15.2 ± 1.1	86.9	**
***git3*Δ**	17.4 ± 1.1	98.3		14.9 ± 0.9	85.1	**
***pka1*Δ**	17.2 ± 1.4	97.2		14.4 ± 0.9	82.3	**
**SPAC27E2.03Δ**	18.0 ± 1.3	101.7		14.4 ± 0.9	82.3	**
***gpa2*Δ**	17.3 ± 1.2	97.7		14.2 ± 0.9	81.1	**
***pyp1*Δ**	17.1 ± 1.5	96.6		13.9 ± 0.6	79.4	**

Table S5. Cell length at division of triple mutants.

Mean cell length at division (in μm) and standard deviation (n=60). ** indicates a *p*-value <0.01 determined by Student's t-test comparing means between each triple mutant and the *cdr1*Δ *sty1*Δ double mutant.

	**Cell length (μm)**	**%**	
***cdr1*Δ *sty1*Δ**	23.0 ± 2.0	100	
***cdr1*Δ *sty1*Δ *ppa2*Δ**	18.7 ± 1.2	81	**
***cdr1*Δ *sty1*Δ *clp1*Δ**	20.4 ± 2.1	89	**
***cdr1*Δ *sty1*Δ *zfs1*Δ**	20.9 ± 1.7	91	**
***cdr1*Δ *sty1*Δ *snf5*Δ**	20.0 ± 1.5	87	**
***cdr1*Δ *sty1*Δ *sol1*Δ**	20.2 ± 1.2	88	**
***cdr1*Δ *sty1*Δ *ski3*Δ**	23.8 ± 2.0	103	
***cdr1*Δ *sty1*Δ *nif1*Δ**	22.5 ± 1.5	98	

Table S6. Genetic interaction between mutations that are additive to both *cdr1*Δ and *sty1*Δ.

Mean cell length at division (in μm) ± s.d. (n=60) is shown. ** indicates significant difference in cell length (*p*-value <0.01) between the double mutant and both single parental mutant strains.

	Cell length (μm)		Cell length (μm)	
**WT**	14.1 ± 0.8	***ppa2*Δ *clp1*Δ**	9.3 ± 0.9	**
***sol1*Δ**	12.9 ± 0.9	***ppa2*Δ *snf5*Δ**	10.3 ± 0.8	**
***clp1*Δ**	11.8 ± 1.1	***ppa2*Δ *zfs1*Δ**	9.6 ± 0.7	**
***snf5*Δ**	12.6 ± 0.7	***sol1*Δ *snf5*Δ**	12.5 ± 0.7	
***ppa2*Δ**	11.3 ± 0.8	***sol1*Δ *ppa2*Δ**	10.6 ± 0.8	**
***zfs1*Δ**	12.3 ± 0.7	***sol1*Δ *zfs1*Δ**	11.4 ± 0.7	**
		***sol1*Δ *clp1*Δ**	10.8 ± 1.7	**
		***zfs1*Δ *clp1*Δ**	10.7 ± 0.9	**
		***clp1*Δ *snf5*Δ**	10.6 ± 0.9	**
		***snf5*Δ *zfs1*Δ**	10.7 ± 0.5	**

Table S7. Cell length at division of mutants carrying a non-phosphorylatable version of the cyclin-dependent kinase Cdc2.

Snf5, *sol1*, *zfs1* and *ppa2* genes were deleted in a strain carrying the Cdc13-L-Cdc2 or Cdc13-L-(T14A Y15F)Cdc2 fusion proteins. In Cdc13-L-(T14A Y15F)Cdc2, the Cdc2 moiety carries mutations Thr14Ala and Tyr15Phe, preventing phosphorylation by the Wee1 and Mik1 kinases. Mean cell length at division (in μm) ± s.d. (n=60 cells, for Cdc13-L-Cdc2 strains; n=150 cells for Cdc13-L-(T14A Y15F)Cdc2 strains). ND, not determined.

	***cdc2*Δ *cdc13*Δ** **Cdc13-L-Cdc2**	**%**	***cdc2*Δ *cdc13*Δ** **Cdc13-L-(T14A Y15F)Cdc2**	**%**
**-**	14.8 ± 0.9	100	12.0 ± 1.7	100
***sty1*Δ**	19.3 ± 1.9	130	13.5 ± 2.4	113
***cdr1*Δ**	20.1 ± 1.3	136	12.1 ± 2.3	101
***snf5*Δ**	13.0 ± 0.7	88	10.4 ± 1.5	87
***sol1*Δ**	13.0 ± 0.9	88	10.6 ± 1.6	88
***zfs1*Δ**	13.1 ± 0.8	89	10.4 ± 2.1	87
***ppa2*Δ**	13.0 ± 0.9	88	Lethal	
***snf5*Δ *sol1*Δ**	ND		10.4 ± 1.0	87
***snf5*Δ *zfs1*Δ**	ND		9.3 ± 1.3	78

Table S8. Strains used in this work.

Name	Genotype	Origin
PN1	972 h-	Lab collection
PN558	leu1-32 ura4-D18 ade6-M210 h+	Lab collection
FR301	pyp1Δ::kanMX6 h-	This work
FR303	clp1Δ::kanMX6 h+	This work
FR309	pka1Δ::kanMX6 h-	This work
FR305	gpa2Δ::kanMX6 h-	This work
FR495	pom1Δ::kanMX6 h+	This work
FR299	snf5Δ::kanMX6 h-	This work
FR297	ppa2Δ::kanMX6 h+	This work
FR376	git3Δ::kanMX6 h+	This work
FR373	git5Δ::kanMX6 h-	This work
FR382	pab2Δ::kanMX6 h-	This work
FR379	ski3Δ::kanMX6 h-	This work
FR370	sgf73Δ::kanMX6 h-	This work
FR424	sol1Δ::kanMX6 h+	This work
FR428	SPAC27E2.03cΔ::kanMX6 h-	This work
FR434	zfs1Δ::kanMX6 h+	This work
FR538	nif1Δ::kanMX6 h+	This work
FR1205	wee1Δ::kanMX6 h+	This work
FR399	SPBC19F8.02Δ::kanMX6 h+	This work
FWP230	snf22Δ::ura4 ura4-D18 leu1-32 ade6-M210 h-	Gift from Fred Winston
FR686	ski3Δ::kanMX6 zfs1Δ::kanMX6 h+	This work
FR1137	ski3Δ::kanMX6 zfs1Δ::kanMX6 ppa2Δ::kanMX6 h-	This work
FR1106	ski3Δ::kanMX6 zfs1Δ::kanMX6 ppa2Δ::kanMX6 snf5Δ::kanMX6 h-	This work
FR1141	ski3Δ::kanMX6 zfs1Δ::kanMX6 ppa2Δ::kanMX6 snf5Δ::kanMX6 clp1Δ::natMX6 h-	This work
FR447	sty1Δ::natMX6 pyp1Δ::kanMX6 h-	This work
FR455	sty1Δ::natMX6 clp1Δ::kanMX6 h-	This work
FR453	sty1Δ::natMX6 pka1Δ::kanMX6 h+	This work
FR583	sty1Δ::natMX6 gpa2Δ::kanMX6 h-	This work
FR443	sty1Δ::natMX6 pom1Δ::kanMX6 h-	This work
FR451	sty1Δ::natMX6 snf5Δ::kanMX6 h+	This work
FR528	sty1Δ::natMX6 ppa2Δ::kanMX6 h-	This work
FR475	sty1Δ::natMX6 git3Δ::kanMX6 h+	This work
FR485	sty1Δ::natMX6 git5Δ::kanMX6 h+	This work
FR910	sty1Δ::natMX6 pab2Δ::kanMX6 h-	This work
FR530	sty1Δ::natMX6 ski3Δ::kanMX6 h-	This work
FR1039	sty1Δ::natMX6 sgf73Δ::kanMX6 h-	This work
FR508	sty1Δ::natMX6 sol1Δ::kanMX6 h+	This work
FR510	sty1Δ::natMX6 SPAC27E2.03cΔ::kanMX6 h-	This work
FR637	sty1Δ::natMX6 zfs1Δ::kanMX6 h+	This work
FR551	sty1Δ::natMX6 nif1Δ::kanMX6 h+	This work
FR449	cdr1Δ::natMX6 pyp1Δ::kanMX6 h+	This work
FR457	cdr1Δ::natMX6 clp1Δ::kanMX6 h+	This work
FR481	cdr1Δ::natMX6 pka1Δ::kanMX6 h-	This work
FR635	cdr1Δ::natMX6 gpa2Δ::kanMX6 h+	This work
FR445	cdr1Δ::natMX6 pom1Δ::kanMX6 h-	This work
FR469	cdr1Δ::natMX6 snf5Δ::kanMX6 h+	This work
FR491	cdr1Δ::natMX6 ppa2Δ::kanMX6 h+	This work
FR493	cdr1Δ::natMX6 git3Δ::kanMX6 h+	This work
FR483	cdr1Δ::natMX6 git5Δ::kanMX6 h-	This work
FR471	cdr1Δ::natMX6 pab2Δ::kanMX6 h-	This work
FR532	cdr1Δ::natMX6 ski3Δ::kanMX6 h+	This work
FR1040	cdr1Δ::natMX6 sgf73Δ::kanMX6 h+	This work
FR506	cdr1Δ::natMX6 sol1Δ::kanMX6 h-	This work
FR512	cdr1Δ::natMX6 SPAC27E2.03cΔ::kanMX6 h-	This work
FR504	cdr1Δ::natMX6 zfs1Δ::kanMX6 h+	This work
FR553	cdr1Δ::natMX6 nif1Δ::kanMX6 h+	This work
FR402	cdr1Δ::natMX6 sty1Δ::natMX6 h-	This work
FR728	cdr1Δ::natMX6 sty1Δ::natMX6 ppa2Δ::kanMX6 h+	This work
FR730	cdr1Δ::natMX6 sty1Δ::natMX6 clp1Δ::kanMX6 h+	This work
FR732	cdr1Δ::natMX6 sty1Δ::natMX6 zfs1Δ::kanMX6 h-	This work

FR1038	cdr1Δ::natMX6 sty1Δ::natMX6 snf5Δ::kanMX6 h-	This work
FR1099	cdr1Δ::natMX6 sty1Δ::natMX6 sol1Δ::kanMX6 h-	This work
FR1232	cdr1Δ::natMX6 sty1Δ::natMX6 sgf73Δ::kanMX6 h+	This work
FR1095	cdr1Δ::natMX6 sty1Δ::natMX6 nif1Δ::kanMX6 h+	This work
FR1097	cdr1Δ::natMX6 sty1Δ::natMX6 ski3Δ::kanMX6 h+	This work
FR1046	sol1Δ::kanMX6 ppa2Δ::kanMX6 h-	This work
FR1131	clp1Δ::natMX6 ppa2Δ::kanMX6 ade6-M21X h-	This work
FR1072	snf5Δ::kanMX6 ppa2Δ::kanMX6 h+	This work
FR690	zfs1Δ::kanMX6 ppa2Δ::kanMX6 h+	This work
FR1051	clp1Δ::kanMX6 sol1Δ::kanMX6 h+	This work
FR602	snf5Δ::kanMX6 sol1Δ::kanMX6 h+	This work
FR1044	zfs1Δ::kanMX6 sol1Δ::kanMX6 h+	This work
FR1070	snf5Δ::kanMX6 clp1Δ::kanMX6 h+	This work
FR1133	zfs1Δ::kanMX6 clp1Δ::natMX6 ade6-M21X h-	This work
FR688	zfs1Δ::kanMX6 snf5Δ::kanMX6 h-	This work
PN10714*	leu1Δ::[P_{cdc13}-cdc13-L-cdc2(T14A Y15F)as-T_{cdc13} ura4+] ura4-D18 cdc2Δ::kanMX6 cdc13Δ::natMX6 cig1Δ::ura4+ cig2Δ::ura4+ puc1Δ::ura4+ h-	[11]
FR735*	leu1Δ::[P_{cdc13}-cdc13-L-cdc2(T14A Y15F)as-T_{cdc13} ura4+] ura4-D18 cdc2Δ::ScLEU2 cdc13Δ::natMX6 h-	This work
FR808*	snf5Δ::kanMX6 leu1Δ::[P_{cdc13}-cdc13-L-cdc2(T14A Y15F)as-T_{cdc13} ura4+] ura4-D18 cdc2Δ::ScLEU2 cdc13Δ::natMX6 h+	This work
FR1027*	sol1Δ::kanMX6 leu1Δ::[P_{cdc13}-cdc13-L-cdc2(T14A Y15F)as-T_{cdc13} ura4+] ura4-D18 cdc2Δ::ScLEU2 cdc13Δ::natMX6 h-	This work
FR841*	zfs1Δ::kanMX6 leu1Δ::[P_{cdc13}-cdc13-L-cdc2(T14A Y15F)as-T_{cdc13} ura4+] ura4-D18 cdc2Δ::ScLEU2 cdc13Δ::natMX6 h+	This work
FR1042*	snf5Δ::kanMX6 sol1Δ::kanMX6 leu1Δ::[P_{cdc13}-cdc13-L-cdc2(T14A Y15F)as-T_{cdc13} ura4+] ura4-D18 cdc2Δ::ScLEU2 cdc13Δ::natMX6 h+	This work
FR862*	zfs1Δ::kanMX6 snf5Δ::kanMX6 leu1Δ::[P_{cdc13}-cdc13-L-cdc2(T14A Y15F)as-T_{cdc13} ura4+] ura4-D18 cdc2Δ::ScLEU2 cdc13Δ::natMX6 h+	This work
FR1243*	cdr1Δ::kanMX6 leu1Δ::[P_{cdc13}-cdc13-L-cdc2(T14A Y15F)as-T_{cdc13} ura4+] ura4-D18 cdc2Δ::kanMX6 cdc13Δ::natMX6 h+	This work
FR1247*	sty1Δ::natMX6 leu1Δ::[P_{cdc13}-cdc13-L-cdc2(T14A Y15F)as-T_{cdc13} ura4+] ura4-D18 cdc2Δ::kanMX6 cdc13Δ::natMX6 h-	This work
PN10657*	leu1Δ::[P_{cdc13}-cdc13-L-cdc2as-T_{cdc13} ura4+] ura4-D18 cdc2Δ::kanMX6 cdc13Δ::natMX6 cig1Δ::ura4+ cig2Δ::ura4+ puc1Δ::ura4+ h+	[11]
FR1107*	leu1Δ::[P_{cdc13}-cdc13-L-cdc2as-T_{cdc13} ura4+] ura4-D18 cdc2Δ::kanMX6 cdc13Δ::natMX6 h+	This work
FR1116*	snf5Δ::kanMX6 leu1Δ::[P_{cdc13}-cdc13-L-cdc2as-T_{cdc13} ura4+] ura4-D18 cdc2Δ::kanMX6 cdc13Δ::natMX6 h+	This work
FR1115*	sol1Δ::kanMX6 leu1Δ::[P_{cdc13}-cdc13-L-cdc2as-T_{cdc13} ura4+] ura4-D18 cdc2Δ::kanMX6 cdc13Δ::natMX6 h+	This work
FR1122*	zfs1Δ::kanMX6 leu1Δ::[P_{cdc13}-cdc13-L-cdc2as-T_{cdc13} ura4+] ura4-D18 cdc2Δ::kanMX6 cdc13Δ::natMX6 h+	This work
FR1140*	ppa2Δ::kanMX6 leu1Δ::[P_{cdc13}-cdc13-L-cdc2as-T_{cdc13} ura4+] ura4-D18 cdc2Δ::kanMX6 cdc13Δ::natMX6 h+	This work
FR1241*	cdr1Δ::kanMX6 leu1Δ::[P_{cdc13}-cdc13-L-cdc2as-T_{cdc13} ura4+] ura4-D18 cdc2Δ::kanMX6 cdc13Δ::natMX6 h-	This work
FR1245*	sty1Δ::natMX6 leu1Δ::[P_{cdc13}-cdc13-L-cdc2as-T_{cdc13} ura4+] ura4-D18 cdc2Δ::kanMX6 cdc13Δ::natMX6 h+	This work
FR878	cdc2:REP5[P_{nmt1}-cdc2-YFP-T_{nmt1} sup3-5] (P_{nmt1}-cdc2-YFP-T_{nmt1})Δ::kanMX6 h-	[12]
FR926	ppa2Δ::kanMX6 cdc2:REP5[P_{nmt1}-cdc2-YFP-T_{nmt1} sup3-5] (P_{nmt1}-cdc2-YFP-T_{nmt1})Δ::kanMX6 h+	This work
FR885	snf5Δ::kanMX6 cdc2:REP5[P_{nmt1}-cdc2-YFP-T_{nmt1} sup3-5] (P_{nmt1}-cdc2-YFP-T_{nmt1})Δ::kanMX6 h+	This work
FR883	zfs1Δ::kanMX6 cdc2:REP5[P_{nmt1}-cdc2-YFP-T_{nmt1} sup3-5] (P_{nmt1}-cdc2-YFP-T_{nmt1})Δ::kanMX6 h+	This work
FR880	zfs1Δ::kanMX6 snf5Δ::kanMX6 cdc2:REP5[P_{nmt1}-cdc2-YFP-T_{nmt1} sup3-5] (P_{nmt1}-cdc2-YFP-T_{nmt1})Δ::kanMX6 h-	This work
FR874	cdc13:REP5[P_{nmt1}-cdc13-YFP-T_{nmt1} sup3-5] h-	[12]
FR926	ppa2Δ::kanMX6 cdc13:REP5[P_{nmt1}-cdc13-YFP-T_{nmt1} sup3-5] h+	This work
FR886	snf5Δ::kanMX6 cdc13:REP5[P_{nmt1}-cdc13-YFP-T_{nmt1} sup3-5] h+	This work
FR889	zfs1Δ::kanMX6 cdc13:REP5[P_{nmt1}-cdc13-YFP-T_{nmt1} sup3-5] h+	This work
FR892	zfs1Δ::kanMX6 snf5Δ::kanMX6 cdc13:REP5[P_{nmt1}-cdc13-YFP-T_{nmt1} sup3-5] h+	This work
PN1682	rum1Δ::ura4+ ura4-D18 h-	Lab collection
FR1171	rum1Δ::ura4+ ppa2Δ::kanMX6 ura4-D18 h+	This work
FR1169	rum1Δ::ura4+ snf5Δ::kanMX6 ura4-D18 h+	This work
FR1167	rum1Δ::ura4+ zfs1Δ::kanMX6 ura4-D18 h+	This work

*, ***as*** refers to F84G substitution in the Cdc2 moiety of the fusion protein, which makes the kinase sensitive to the ATP-analog NmPP1.

References

1. Aoyama K, Mitsubayashi Y, Aiba H, Mizuno T: **Spy1, a histidine-containing phosphotransfer signaling protein, regulates the fission yeast cell cycle through the Mcs4 response regulator.** *J Bacteriol* 2000, **182:**4868-4874.
2. Petersen J, Nurse P: **TOR signalling regulates mitotic commitment through the stress MAP kinase pathway and the Polo and Cdc2 kinases.** *Nat Cell Biol* 2007, **9:**1263-1272.
3. López-Avilés S, Grande M, González M, Helgesen A-L, Alemany V, Sanchez-Piris M, Bachs O, Millar JBA, Aligue R: **Inactivation of the Cdc25 phosphatase by the stress-activated Srk1 kinase in fission yeast.** *Mol Cell* 2005, **17:**49-59.
4. Nefsky B, Beach D: **Pub1 acts as an E6-AP-like protein ubiquitiin ligase in the degradation of cdc25.** *EMBO J* 1996, **15:**1301-1312.
5. Ford JC, al-Khodairy F, Fotou E, Sheldrick KS, Griffiths DJ, Carr AM: **14-3-3 protein homologs required for the DNA damage checkpoint in fission yeast.** *Science* 1994, **265:**533-535.
6. Calonge TM, Eshaghi M, Liu J, Ronai Ze, O'Connell MJ: **Transformation/transcription domain-associated protein (TRRAP)-mediated regulation of Wee1.** *Genetics* 2010, **185:**81-93.
7. Gilbreth M, Yang P, Bartholomeusz G, Pimental RA, Kansra S, Gadiraju R, Marcus S: **Negative regulation of mitosis in fission yeast by the Shk1 interacting protein Skb1 and its human homolog, Skb1Hs.** *PNAS* 1998, **95:**14781-14786.
8. Kim MJ, Park EJ, Park SD: **A glucose-inducible gene in *Schizosaccharomyces pombe*, *rrg1*$^{+}$, is involved in negative regulation of G2/M progression.** *Mol Cells* 2002, **14:**312-317.
9. Jorgensen P, Nishikawa JL, Breitkreutz BJ, Tyers M: **Systematic identification of pathways that couple cell growth and division in yeast.** *Science* 2002, **297:**395-400.
10. Zhang J, Schneider C, Ottmers L, Rodriguez R, Day A, Markwardt J, Schneider BL: **Genomic scale mutant hunt identifies cell size homeostasis genes in *S. cerevisiae*.** *Curr Biol* 2002, **12:**1992-2001.
11. Coudreuse D, Nurse P: **Driving the cell cycle with a minimal CDK control network.** *Nature* 2010, **468:**1074-1079.
12. Decottignies A, Zarzov P, Nurse P: **In vivo localisation of fission yeast cyclin-dependent kinase cdc2p and cyclin B cdc13p during mitosis and meiosis.** *J Cell Sci* 2001, **114:**2627-2640.
